# Evaluation of Clinical Phenomena in Psychiatry

**Published:** 1969-10

**Authors:** J. Crawford Little


### THE EVALUATION OF CLINICAL PHENOMENA IN PSYCHIATRY

**J. Crawford Little**

The problems which I wish to discuss today are those associated with the observing and eliciting of signs, and with the assessing and eliciting of symptoms in psychiatric patients. Whatever diagnosis we arrive at, whatever structure of psychodynamics we may subsequently erect in our speculative attempts to explain our patient's condition, these intellectual exercises must arise from what we or others have observed about the patient, and from what the patient communicates.

Despite everything else that Professor Perry has contributed to medical science, to teaching and to administration, on me personally (and I am sure very many of his ex-students would agree) the most lasting influence has been his persevering insistence on the absolutely fundamental importance of good history-taking and of a thorough and decisive examination of the patient. He detested evasions and fence-sitting, and if, like all effective undergraduate clinical teachers, he didactically over-simplified the complex perceptual issues involved, we forgive him. Not for us the posture of the obsessional patient who when asked "Are you a decisive person?" replied after lengthy cogitation —"Er . . . yes and no ! "

And so, with this introduction in mind, I offer a humorous pictorial tribute to Professor Perry, the original of which, some twenty-five years ago, was intended I think for the Black Bag but never indeed, until now, has seen the light of day; and little did I guess that such an opportunity would ever arise.*

THE NEED FOR DIAGNOSIS

It is accepted medical teaching that diagnosis precedes treatment, that before taking action we assess the characteristics of the maladjustment and judge how closely the sign-symptom complex in its development approximates to certain disease-concept models. A major problem which has bedevilled epidemiological and therapeutic comparative studies in psychiatry is the uncertainty and controversy as to what these models should comprise. There is such widespread dissatisfaction with traditional diagnostic classification that many psychiatrists, particularly across the Atlantic, prefer to dispense with this step altogether, concentrating instead on attempts to " understand " the apparent psychodynamics on the grounds that every patient is unique, which indeed he is. However, no medical practice with pretensions to scientific acceptance can proceed on this basis, and even the most ardent psychodynamicist, whether he be aware of his logical manipulations or not, in fact follows Freud, who, as Roth (1967) points out, *separately* studied and described the psychodynamics of hysteria, phobias, obsessions, melancholia, paranoid states and homosexual deviation. Indeed, definition and classification are necessary prerequisites to the study of mechanisms in all scientific enquiry. Good science, like good art, creates order out of chaos.

Despite the impression carried away by some trainees in psychiatry that the teaching case-conference pursues diagnostic classification as an end in itself, I believe such basic training to be fundamental; what we require now is not the abandonment of diagnostic pattern-making, but its refinement. The topic is in the forefront of debate; is it to be "first-aid or euthanasia" for

*The illustration referred to appears opposite p. 105. Eds.

psychiatric classification as Dohrenwend (1969) arrestingly points the choice?

Diagnosis is only a means to an end, however comforting the closure of the Gestalt may be to the practitioner. Psychologists describe the primitive mental phenomenon of "nominal realism", the belief that power and control over a potentially threatening person or object can be gained by naming it, exemplified in psychiatry by the "don't just do something—stand there!" school.

A good diagnostic decision will group our immediate patient with others presenting similar manifestations, so that from our training, experience, and reading we may hazard a more likely estimate of what will happen if events are allowed to continue—the natural prognosis; and of what may happen if we attempt to interfere in various ways with beneficent intent—the treatment prospects. Additionally, or alternatively, we may gain knowledge as to how our patient, as a representative of the group, arrived at his present maladjusted state—ætiology; and just possibly plan what we might do to reduce the possibility of relapse—secondary prevention; or what we might do or advise for others heading along the same path, long before breakdown point is reached—primary prevention.

Thus to summarize: diagnosis is no sterile intellectual exercise but a pointer to what has gone wrong and what we might do about it; a good classificatory system must have practical validity.

### DIAGNOSTIC CONFORMITY

For a number of years now very serious efforts have been made on a national and international scale to improve diagnostic conformity, for, as Feinstein (1967) affirms, the critical quality of scientific data is not accuracy but reproducibility. In this country a distinguished committee of psychiatrists, epidemiologists, and statisticians has prepared for the General Register Office a Glossary of Mental Disorders (1968), containing precise descriptions of each category, and designed to complement the eighth (1965) edition of the International Classification of Diseases. The glossary is a very real step on the right road, but its use in our hospital for the past six months already suggests that all users should accept the invitation of the innovators to communicate criticisms and suggestions in time for the 1975 edition. However, even with this imperfect instrument Shepherd *et al* (1968) have demonstrated encouraging levels of inter-observer diagnostic agreement in an international study using prepared written case-histories and video-taped clinical interviews, and not surprisingly, have shown that agreement was highest where the cases were most "typical" in respect of reported history and mental status on examination. The exercise suggests that some form of *multidimensional* system of classification will eventually be required for statistical purposes as an advance on the over-simplified "principal" and "other" single diagnostic statements demanded by the conventions of usage of the I.C.D.

### SYMPTOMS, SIGNS, AND PERCEPTUAL EXPECTATIONS

I would now like to turn to the assessment of symptoms and observation of signs, which together form the basis for our diagnostic formulations, and I would emphasize the formidable problems of observer bias and concept-driven perception involved.

Epidemiological studies have indicated that the apparent incidence of schizophrenia in the United States is approximately fifty per cent higher than that detected in England and Wales as reflected in age-adjusted first admission rates (Kramer 1961). If this is a "true" difference the finding would have considerable ætiological significance. But have we here a spurious difference reflecting differing national diagnostic concepts or nomenclature, quite apart from possible social influences affecting admission to hospital, which one feels must contribute to the ninefold first admission rate for manic-depressive psychosis in England and Wales? A general medical audience will recall the puzzling high incidence of chronic emphysema in the U.S. in comparison with the excessive amount of chronic bronchitis in the U.K., a riddle only resolved when international co-operation revealed that one and the same condition was being described under these different names.

With respect to schizophrenia, British psychiatrists were impressed by the apparent adoption of much less rigid criteria by U.S. psychiatrists in making this diagnosis, but a recent study by Hordern *et al.* (1968) reveals a curious perceptual mechanism underlying this discrepancy, in that both U.S. and U.K. psychiatrists were shown to adopt a similar rank-order of signs and symptoms required to establish the diagnosis of schizophrenia, and indeed of many other psychiatric disorders. The authors conclude "psychiatrists find it easier to envisage, describe and agree on the theoretical attributes of a category of psychiatric illness than to agree on the diagnostic category a particular patient should occupy." The perceptual process of emphasis of some, and elimination of other observable signs and symptoms varies between the two national professional groups. Herein lies the hazard: one starts off prepared to see what one expects and what authoritarian teaching has led one to select.

The difficulties can be illustrated by an anecdote and a study, both culled from the literature.

Lehmann (1967) instances a resident who diagnosed schizophrenia appropriately enough on the presence of Bleuler's four primary symptoms—autism, blunted affect, loosening of associations, and ambivalence, plus, for good measure, bizarre behaviour. Lehmann himself interviewed the patient and regarded the "autistic" withdrawal as merely tension and reserve, the "ambivalence" was the patient's reasonable uncertainty whether to stay in hospital or go home, the "blunted" mood state was simply the Latin-American resident's assessment of the Anglo-Saxon patient's emotional control, the "loosening of associations" was a failure to interpret a proverb precisely, and the "bizarre" behaviour a matter of cultural differences. In the absence, except in certain organic states, of any appeal to more objective evidence from the laboratory or P.M. room, who then is "right" and who is "wrong"? I hope to indicate later why the question in this form can have no answer, but that a legitimate alternative should take its place.

Temerlin (1968) reports from Oklahoma a study of considerable interest to those impressed by the influence of authoritarian opinion. A tape-recorded interview with a normal subject was played over to a group of psychiatrists who were asked independently to give an opinion, having been told beforehand "two board-certified psychiatrists . . . had found the recording interesting because the patient looked neurotic but actually was quite psychotic . . ." In the event fifteen psychiatrists considered the subject to be psychotic, ten opted for neurosis and character disorder, while none thought the subject mentally healthy. In a control group not given such suggestion none diagnosed psychosis. The author suggests that in medicine there is a tendency to play safe and diagnose illness when in doubt, and further that the system may reward conformity with prestige figures. (I would agree: before age thirty-five we dare not rebel—after forty-five we cannot). Temerlin comments that despite implicit instructions to avoid inferences and to be descriptive, only the few (non-psychiatrists) who diagnosed health had done so. Most subjects either mixed inferences and observations, or reported inferences exclusively; some reported inferences labelled as observations.

### THE MYTH OF OBJECTIVITY

To study signs and symptoms in order to establish a tentative diagnosis, and then to attempt its refutation by further critical observation, is, in essence, identical with the general scientific method of testing a hypothesis. For many years, without being formally aware of it, I held the Baconian view, which Newton professed, that the investigator makes his observations with the eye of innocence, sorts the data logically in his mind, and proceeds to induce generalisations therefrom. However, Popper (1959, 1963) for forty years has been a formidable opponent of any inductivist or positivist methodology, and has argued his case, first in his book "The Logic of Scientific Discovery", and subsequently in a large number of papers most of which are collected in his volume "Conjectures and Refutations." Medawar (1967) has expanded this viewpoint most forcibly in his book "The Art of the Soluble," and I, with many others, am won over to the view that there is no such thing as "innocent" observation, but that, to quote Whewell (1847) "there is a mask of theory over the whole face of nature". Thus the investigator's perceptions are always driven by his expectations, so that the reviewer who complained that Churchill's " History of the English Speaking Peoples " should have been entitled "What Interests Churchill in History" was expressing a generalisation about all historians, which for our purpose includes the patient and his doctor.

I would emphasize that the perceptual problem which faces the psychiatrist is common to all observers of natural phenomena, and is particularly acute in the behavioural field, rather less so in the biological sciences, and at its least disturbing in the physical sciences—hence the general desire of the scientifically-minded clinician to escape from the patient into the laboratory.

In clinical medicine no one has felt the same since Fletcher (1952) demonstrated alarming discrepancies in the independent assessments made by eight experienced physicians of twelve key signs in pulmonary emphysema (directly observable signs, not symptoms which require assessment). Studies of similar disagreement in observation of other physical signs are well known, as are those of radiologists' and endoscopists' differing perceptions and interpretations. Less well recognised perhaps are histologists' differences of opinion about about the differentiation of inflammation and neoplasm, of benign and malignant tumours and of the histological types of cancer (Feinstein 1967).

### CLINICAL JUDGMENT

The psychiatrist is deprived, for better or for worse, of the help of morbid anatomists, chemical pathologists, micro-biologists, endoscopists, radiologists, and laparotomists. He is in truth the last of the clinicians, the only doctor who attempts a comprehensive history, and relies solely on the "evidence" of his trained senses and feelings. Can he rely on his visual and auditory emotion-driven perceptions, or is he just deceiving himself all the time? What he has to observe is frequently so subtle and complex as to defy precise definition, let alone measurement, and the more valuable the descriptive category the greater seems to be the difficulty. His most developed diagnostic perceptual capacity, much neglected in medical training, is his ability to " tune in " or emphathize with the emotional state of his patient, and how objective is that?

I should like to end on a more optimistic note and suggest that the issues are not as clouded and impossible as might seem from what I have said. Platt (1969) has written "I have no more difficulty in recognising a willow tree than I would have in recognising a friend in Trafalgar Square. I don't require a techinician to take 24 hour samples of sap and send me a report in a week's time which tells me that the chances of this being a willow tree are significantly higher than its being any other tree." Does a doctor need a technician to tell him his patient is answering past the point and shows a degree of emotional incongruity?

Not only have psychiatrists been investigating levels of agreement in diagnosis but they have also been giving considerable attention to the basic signs and symptoms, the detection and assessment of which form the foundation on which the diagnosis is built (*pace* popper).

I have recently become involved in the use of the standardized schedule for measuring and classifying "Present Psychiatric State" evolved by the M.R.C. Social Psychiatry Research Unit at the Maudsley Hospital in connection with the U.S.-U.K. Diagnostic Project (Wing *et al.*, 1967; Kendall *et al.*, 1968). As in all standardized procedures there is a certain loss of information, but this venture (now in its eighth revision) offers an approach more flexible than most of its predecessors and rivals. I have already emphasized that it is reproducibility that converts a private observation into a public one and hence renders it susceptible to scientific manipulation (Little, 1968), and it is certainly encouraging that among those trained in its use the levels of independent observer agreement in this exhaustive 63-page schedule covering 500 ratings are higher than we might have dared to hope. The results are superior to those of a previous study by Kreitman *et al.* (1961) in which diagnostic implications were overtly allowed to bias detailed perception of signs and symptoms.

The five hundred items in the Present State Examination were grouped into forty-eight sections, each dealing with a more or less well-defined area of psychopathology. In twenty-three of the sections covering the main part of the interview and most of the commoner areas of psychopathology, the average product moment correlation between observers was 0.84, and only two sections had a correlation below 0.70. "Quite a lengthy training period and a series of joint interviews with an experienced rater are usually necessary before a new rater masters the conventions of the interview and becomes fluent in its administration."

Phenomenological psychopathology, in the sense of the study of abnormal psychic phenomena *as such,* their formal aspects, apart from their meaning, has in Hoenig's (1968) opinion been much neglected, but English translations of Jasper's (1923) and Schneider's (1959) books are now available. There is the appropriate section of the text book by Kräupl Taylor (1966) and Fish's (1967) small text on the subject will be found most useful.

### CONCLUSION

My subject has been the evaluation of clinical phenomena in psychiatry, and I have indicated that with respect to both signs and symptoms (the latter particularly), and diagnosis, progress is at last being made in an attempt to estimate the degrees of conformity achievable, and to scrutinize possible reasons for discrepancies and determine whether these be due to differences in perception, in interpretation, or in descriptive language or nosological usage. Much work remains to be done, but the results so far are encouraging for those who hold that the patient, his experiences and sufferings, and the natural history of his dis-ease are the true concern of the physician, and that science need not go out of the window as the clinician enters the room.

The idea of total objectivity is a myth, whatever the observation being made. Once we appreciate the full extent of the ever present phenomenon of perceptual set induced by theoretical expectations we are to that extent freed to appreciate unsuspected relationships and possibly achieve a fresh vision of events and processes, always accepting the inherent danger that this in turn may become the new conventional wisdom which, over-taught to our juniors, may freeze progress for a further generation.

Despite the efforts I have outlined much of psychiatric practice remains, and will remain, intuitive. Many of the problems presented by patients and the manner in which we as doctors manage them, defy close analysis, definition, and measurement. Many of the best practising psychiatrists—the ones you would seek out for your family—have little interest in scientific method, utilizing instead their experience, compassion, and commonsense. Good training is vital, but basically the good psychiatrist is born, not made. Among medical students those inclined towards a psychiatric career display certain personality characteristics (Walton *et al.* 1963 and 1964; Walton 1969) which, if not inherent, are probably fixed by this stage of personality evolution.

You might well be puzzled as to just where I stand. Am I now playing the rôle of intuitive therapist or scientific clinician? I leave the question open, for, like Meehl (1954), I have "deliberately left the document in this discursive form to retain the flavour of the mental conflict that besets most of us who do clinical work but try to be scientists."

### REFERENCES

Dohrenwend, B. P. (1969). American Journal of Psychiatry Suppl. to *125*, No. 10.

Feinstein, A. R. (1967). Clinical Judgment. Baltimore, Williams and Wilkins.

Fish, F. (1967). Clinical Psychopathology. Signs and Symptoms in Psychiatry. Bristol, Wright & Son.

Fletcher, C. M. (1952). Proceedings of the Royal Society of Medicine, *45*, 577.

General Register Office (1968): a Glossary of Mental Disorders. (Studies on Medical and Population Subjects No. 22) London. H.M.S.O.

Hoenig, J. (1968). British Journal of Psychiatry, *114*, 895.

Hordern, A., Sandifer, M. G. Green, L. M., and Timbury, G. C. (1968). British Journal of Psychiatry *114*, 935.

Jaspers, K. (1923). General Psychopathology. English translation, J. Hoenig and M. W. Hamilton (1963). Manchester. Manchester Univ. Press.

Kendell, R. E., Everett, B., Cooper, J. E., Sartorius, N., and David, M. E. 1968). Social Psychiatry *3,* 123.

Kramer, M. (1961) in Proceedings of the Third World Congress of Psychiatry. *3,* 153. Montreal, University of Toronto Press/McGill University Press.

Kreitman, N., Sainsbury, P., Morrisey, J., Towers, J., and Scrivener, J. (1961). Journal of Mental Science, *107,* 887.

Lehmann, H. E. (1967). Comprehensive Psychiatry, *8,* 265.

Little, J. C. (1968). Lancet *2,* 1072.

Medawar, P. B. (1967). The Art of the Soluble. London, Methuen.

Meehl, P. E. (1954). Clinical vs. Statistical Prediction. Minneapolis. Univ. of Minnesota Press.

Popper, K. R. (1959). The Logic of Scientific Discovery. London, Hutchinson.

Popper, K. R. (1963). Conjectures and Refutations. London, Routledge and Kegan Paul.

Platt, Lord (1969). British Medical Journal, *1,* 636.

Roth, M. (1967). Comprehensive Psychiatry, *8,* 427.

Schneider, K. (1959). Clinical Psychopathology. English translation, M. W. Hamilton. New York. Grune & Stratton.

Shepherd, M., Brooke, E. M., Cooper, J. E., and Lin, T. (1968). Acta Psychiatrica Scandinavica. Suppl. 201.

Taylor, F. K. (1966). Psychopathology: Its Causes and Symptoms. London. Butterworth.

Temerlin, M. K. (1968). Journal of Nervous and Mental Disease, *147,* 349.

Walton, H. J., Drewery, J., and Carstairs, G. M. (1963). British Medical Journal, *2,* 588.

Walton, H. J., Drewery, J., and Phillip, A. E. (1964) ibid. 2.744.

Walton, H. J. (1969). British Journal of Psychiatry, *115,* 211.

Whewell, W. (1847). The Philosophy of the Induction Sciences, London.

Wing, J. K., Birley, J. L. T., Cooper, J. E., Graham, P., and Isaacs, A. D. (1967). British Journal of Psychiatry, *113,* 499.

